# Application of Generalized Split Linearized Bregman Iteration algorithm for Alzheimer's disease prediction

**DOI:** 10.18632/aging.103017

**Published:** 2020-04-05

**Authors:** Weimin Zheng, Bin Cui, Zeyu Sun, Xiuli Li, Xu Han, Yu Yang, Kuncheng Li, Lingjing Hu, Zhiqun Wang, Alzheimer's Disease Neuroimaging Initiative

**Affiliations:** 1 Department of Radiology, Aerospace Center Hospital, Beijing 100049, China; 2 Deepwise AI lab, Beijing 100080, China; 3 Beijing Huading Jialiang Technology Co, Beijing 100000, China; 4 Department of Radiology, Xuanwu Hospital of Capital Medical University, Beijing 100053, China; 5 Yanjing Medical College, Capital Medical University, Beijing 101300, China

**Keywords:** Alzheimer's disease, machine learning, generalized split linearized Bregman iteration, voxel-based structural magnetic resonance imaging, feature selection

## Abstract

In this paper, we applied a novel method for the detection of Alzheimer’s disease (AD) based on a structural magnetic resonance imaging (sMRI) dataset. Specifically, the method involved a new classification algorithm of machine learning, named Generalized Split Linearized Bregman Iteration (GSplit LBI). It combines logistic regression and structural sparsity regularizations. In the study, 57 AD patients and 47 normal controls (NCs) were enrolled. We first extracted the entire brain gray matter volume values of all subjects and then used GSplit LBI to build a predictive classification model with a 10-fold full cross-validation method. The model accuracy achieved 90.44%. To further verify which voxels in the dataset have greater impact on the prediction results, we ranked the weight parameters and obtained the top 6% of the model parameters. To verify the generalization of model prediction and the stability of feature selection, we performed a cross-test on the Alzheimer's Disease Neuroimaging Initiative (ADNI) and a Chinese dataset and achieved good performances on different cohorts. Conclusively, based on the sMRI dataset, our algorithm not only had good performance in a local cohort with high accuracy but also had good generalization of model prediction and stability of feature selection in different cohorts.

## INTRODUCTION

Alzheimer’s disease (AD) is a progressive neurodegenerative disease leading to dementia, typically manifesting as memory disturbance, attentional and executive deficits, and visuospatial and perceptual impairments. It is pathologically characterized by the deposition of amyloid-β plaques and tau-related neurofibrillary tangles, resulting in loss of neurons [1]. Currently, AD is still an irreversible condition, and there are no effective medications available today. Studies have found that early diagnosis and administration of drugs can delay the progression of the disease. However, it is difficult to diagnose AD in the early stage in clinical practice. Thus, quantitative analysis based on imaging can possibly provide a potential method to make an early diagnosis of AD.

In the past several years, many neuroimaging studies have been performed to develop different biomarkers for the early diagnosis of AD at the individual level [2, 3]. Among various neuroimaging modalities, structural magnetic resonance imaging (sMRI) is most commonly used, possibly due to its wide operability and objectivity. AD pathological changes are mostly involved in the hippocampus, medial temporal gyrus, posterior cingulate gyrus, as well as precuneus [4–7]. Progressive brain atrophy due to neuropathology is often measured using sMRI and is taken as a valuable imaging biomarker for early individual diagnosis of AD [8, 9]. Many neuroimaging studies have used region-of-interest (ROI)-based analysis to explore the subtle local atrophy caused by AD, thus proposing imaging classifiers to distinguish AD from normal control (NC) individuals [10, 11]. Such studies relied solely on prior knowledge to guide the selection of ROI and features, thus ignoring the structural changes of the entire brain and the microstructural abnormalities in the anatomy, which made it difficult and challenging to establish reliable markers for diagnosing AD in the early stages. The potential considerations of classification in clinical practice have largely driven the development of machine learning, which can provide a systematic approach to developing complex, automated, and objective classification frameworks for analyzing high-dimensional data across the whole brain. Typically, the classification framework includes feature extraction and classification algorithms to build predictive models and develop imaging markers to perform classification with high sensitivity and specificity. Applying different classification algorithms on the extracted neuroimaging features for AD/mild cognitive impairment (MCI) showed great advantages for detecting AD at the prodromal stages, even before clinical manifestation [12, 13].

For AD classification, machine learning has attracted increasing attention by using the multimodal quantify patterns of atrophy together with different algorithms in recent years. For feature extraction, brain atrophy was most often quantified via tissue density maps, volume maps, cortical thickness measures, and geometric measures of the hippocampus from sMRI. However, several different classification algorithms have been proposed and applied for AD classification and have achieved promising results. Support vector machine (SVM) is the most popular algorithm for AD classification [14–18]. SVM can extract high-dimensional, informative features from MRI to build predictive classification models, resulting in the automation of clinical diagnosis. Multi-kernel learning, which is an extension of ordinary kernel-based classification algorithms, has also been increasingly used in AD classification [19–21]. Other less common classification algorithms used in AD research include linear discriminant analysis (LDA) [22, 23], orthogonal partial least square regression [24], random forest [25], regularization-based methods [26], voting-based ensemble methods [27], kernel SVM decision-tree [28], and spatially augmented linear programming boosting method (LPBM) [29]. Although several classification algorithms have been applied in AD, there were still some issues to be noted. For example, the algorithms with a general loss and L2 penalty, which could automatically select or extract classification-related features, were poorly interpretable or easy to overfit. Some other algorithms, such as L1 regularization-based methods, which had structural sparsity and interpretability, were inclined to ignore the effect of procedural bias on the classification. Moreover, in our recent study of AD, we found that the preprocessing steps could introduce one type of feature called procedural bias [30], referring to mistakenly enlarged gray matter volume during registration and segmentation in the preprocessing steps. Therefore, for neuroimaging-based AD classification, it has attracted increasing attention to derive a more precise classification algorithm that could take both lesion features and procedural bias into consideration and make better predictions on an individual basis.

Here, to achieve accurate classification of AD, we proposed a new classification algorithm in our preliminary study [30], named the Generalized Split Linearized Bregman Iteration (GSplit LBI) method, which combines logistic regression and structural sparsity regularizations to verify its capacity in a Chinese cohort. The model had a sparsity enforcement based on the idea of variable splitting; therefore, it can effectively leverage both procedural bias and lesion features into prediction, and it has a better regularization path and interpretability. By using this algorithm on the Alzheimer's Disease Neuroimaging Initiative (ADNI) dataset in our previous study, the advantages of GSplit LBI were verified by the improved stability of selected lesion features and better classification results when compared with other algorithms, such as Maximum uncertainty Linear Discriminant Analysis (MLDA), SVM, Lasso, Graphnet, Elastic Net, Total Variation (TV + *l*_1_) and Nonnegative Generalized Fused Lasso (*n*^2^GFL). However, further analysis of the GSplit LBI in AD was not performed; therefore, the key brain regions that determine the classification of AD and its association with clinical variables remain unknown. Most importantly, the generalization and stability of the GSplit LBI model are not very clear. Furthermore, other than the ADNI dataset, we do not know the prediction capacity of the model in other cohorts.

In the present study, we aim to use gray matter (GM) voxels and the GSplit LBI algorithm to distinguish AD from NC at the individual level in a Chinese cohort. We hypothesized that this classification was driven by a distributed pattern of GM voxel alterations that were involved in the temporal lobe, such as the entorhinal cortex, hippocampus, parahippocampal gyrus and other limbic system components. Furthermore, because structural changes might be affected in some specific cognitive-related regions early in the disease course, we expected that atrophy of specific regions could accurately describe and track disease progression, which could be applied as a valuable imaging biomarker for the early diagnosis of AD. In addition, we further extracted these specific regions and made correlations with cognitive performance as measured by mini-mental state examination (MMSE). Finally, we performed a cross-test on the ADNI dataset and our in-house dataset using the model parameters trained from our in-house dataset and the ADNI dataset, respectively. We speculated that the GSplit LBI algorithm not only had good performance in a local cohort but also had generalization of model prediction and stability of feature selection in different cohorts.

## RESULTS

### Demographic and neuropsychological tests

The demographic characteristics are shown in Table 1. There were no significant differences of gender, age and education between the AD and NC groups (both *P* > 0.01). However, the AD group exhibited significantly lower MMSE than the NC group (*P* < 0.0001).

**Table 1:** Characteristics of AD patients and normal controls.

**Characteristics**	**AD**	**Contrlos**	***P* value****
N (M/F)	57 (25/32)	47 (23/24)	0.61^a^
Age, years	65.21±9.14	63.94±8.06	0.46^b^
Education, years	10.56±6.47	11.36±3.59	0.43^b^
MMSE	14.02±5.90	26.49±4.02	<0.0001^b^
CDR	1.43±0.54(1-3)	0	-

MMSE, Mini-Mental State Examination; CDR, Clinical Dementia Rating; plus–minus values are means±S.D.

a The P value for gender distribution in the two groups was obtained by Chi-square test.

b The P values were obtained by a two-sample two-tailed t-test.

### Classification results for an in-house test dataset

**Figure 1:**
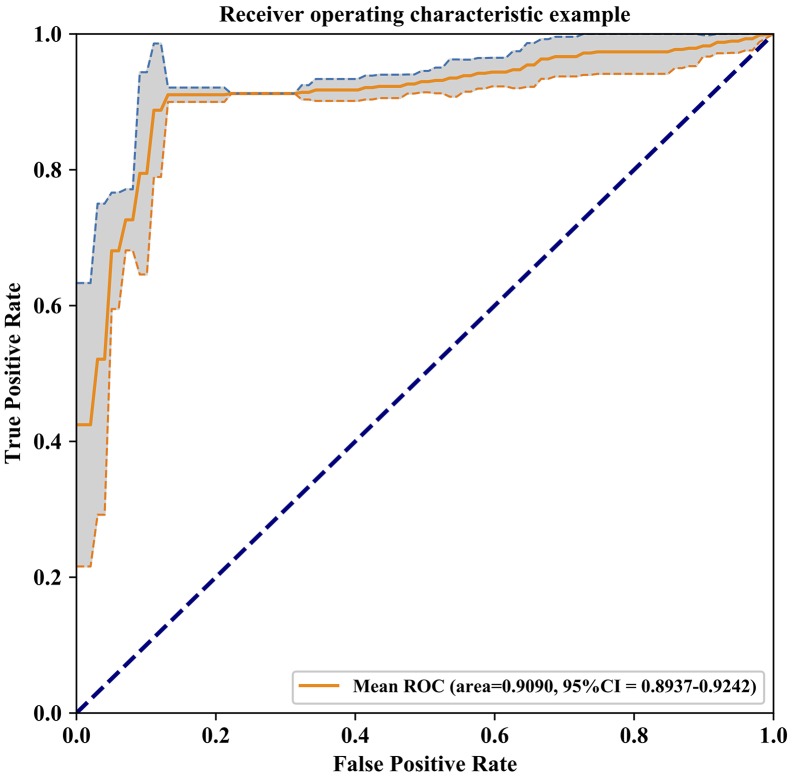
**Receiver Operating Characteristic curve of the model prediction results, The AUC value of our AD/NC classification model is 0.9090 and 95% confidence interval is 0.8937-0.9242 (gray area in the graph).**

After the 10-fold cross-validation was completed, we performed statistical analyses on the results of our model between 57 AD patients and 47 NCs and reported the results of each 10-fold cross-validation in Supplementary Table 1. The overall accuracy, which is the ratio of the correct classification number of AD/NC samples to the total number of AD/NC samples, was 90.44%. In ten cross-validations, the maximum accuracy was 91.36%, and the minimum accuracy was 89.55%. Moreover, the average of sensitivity was 91.17%, ranging from 89.67% to 91.67%, and the average of specificity was 89.50%, ranging from 87.00% to 92.00%. The area under the curve (AUC) value of our AD/NC classification model could reach 0.9090, and the receiver operating characteristic (ROC) curve is shown in Figure 1.

### Brain areas involved in the classification analysis

**Figure 2:**
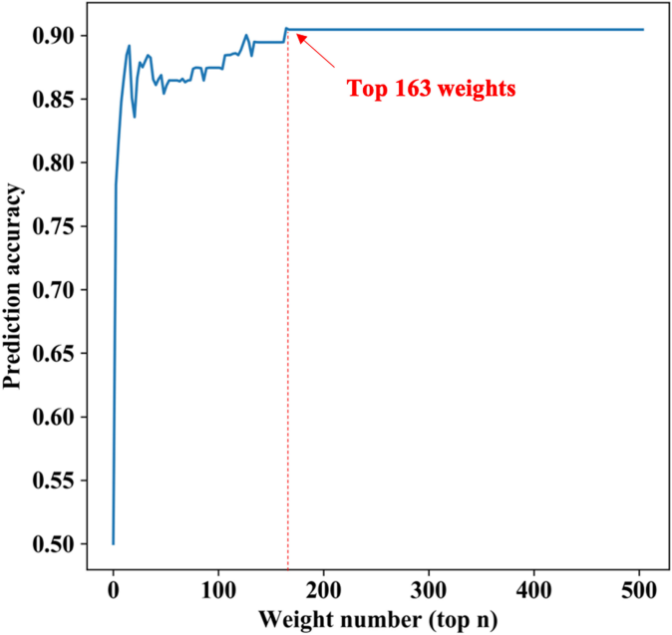
**The relationship between the predicted results and the number of weights.** The horizontal axis represents the largest top n weight parameters, the longitudinal axis represents the prediction accuracy under these parameters. At the beginning of the curve, with the increase of the weight number, the prediction accuracy increases gradually. However, when the largest top parameters number reaches 163 (about the top 6%), the prediction accuracy has the maximum and remains unchanged after that.

**Figure 3:**
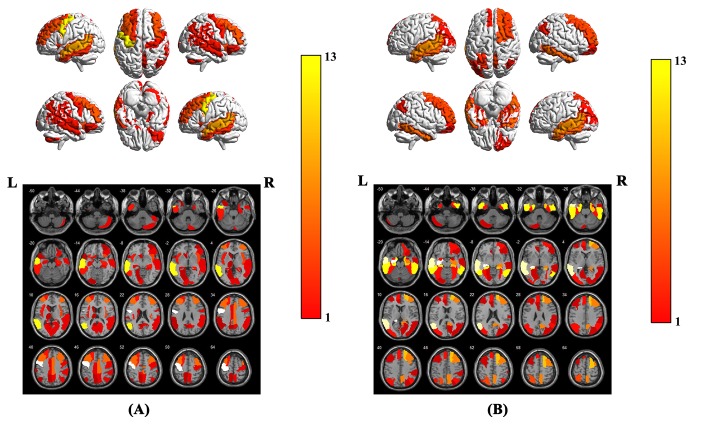
Brain region weight map based on AAL116 template, (**A**) represents the brain regions that have the greatest impact on the classification between AD patients and NCs when using the model parameters of this experiment on our in-house dataset, (**B**) represents the brain regions that have the greatest impact on the classification between AD patients and NCs. The weight value of each brain region is based on the average of the weight values in the brain region. The color bar represents the average weight value in each brain region, the larger the weight value of the model, the warmer the color in the graph.

To verify which voxels in the data have greater impacts on the prediction results, we sorted the weight parameters in main parameter β from large to small and performed classification experiments with the largest top n (ranging from 1 to 2527; 2527 is the number of all input features) weight parameters in turn. We then computed the relationship between the prediction accuracy and the weight parameters used in experiments. As shown in Figure 2, at the beginning of the curve, with the increase of the weight number, the prediction accuracy increased gradually. However, when the weight number reached 163 (about the top 6%), the prediction accuracy attained its maximum and remained unchanged after that. This meant that adding more weight parameters would only produce information redundancy and could not improve performance. Therefore, we defined the voxels corresponding to these top 6% weight parameters as key voxels. The weight distribution map of these voxels is shown in Supplementary Figure 1. The brain regions with the largest weight ratio of our in-house dataset are shown in Figure 3A, and their information is reported in Table 2, including lateral temporal lobe, entorhinal cortex, the hippocampus, parahippocampal gyrus and the limbic system, motor cortex area (M1), cerebellum crus2 and thalamus.

**Table 2:** The brain regions that have the greatest impact on the classification between AD and NC.

**Brain region**	**AAL index**	**Volume(mm^3^)**	**Talairach**			**Weight (%)**
			**X(mm)**	**Y(mm)**	**Z(mm)**	
Left precentral gyrus	1	28174	-38.65	-5.68	50.94	13.25
Left middle temporal gyrus	85	39353	-55.52	-33.80	-2.20	8.77
Right middle frontal gyrus	8	40374	37.59	33.06	34.04	4.80
Left middle frontal gyrus	7	38722	-33.43	32.73	35.46	4.80
Right median cingulate and paracingulate gyri	34	17442	8.02	-8.83	39.79	4.70
Right insula	30	14128	39.02	6.25	2.08	4.34
Left inferior temporal gyrus	89	25647	-49.77	-28.05	-23.17	2.83
Left insula	29	15025	-35.13	6.65	3.44	2.72
Left superior frontal gyrus, dorsolateral	3	28915	-18.45	34.81	42.20	2.68
Right hippocampus	38	7606	29.23	-19.78	-10.33	2.63
Right supramarginal gyrus	64	15770	57.61	-31.50	34.48	2.50
Right superior temporal gyrus	82	25258	58.15	-21.78	-6.80	2.45
Right angular gyrus	66	14009	45.51	-59.98	38.63	2.40
Right middle temporal gyrus	86	35484	57.47	-37.23	-1.47	2.39
Right parahippocampal gyrus	40	9028	25.38	-15.15	-20.47	2.37
Right inferior frontal gyrus, orbital part	16	13747	41.22	32.23	-11.91	2.36
Right precentral gyrus	2	27058	41.37	-8.21	52.09	2.20
Left calcarine fissure and surrounding cortex	43	18157	-7.14	-78.67	6.44	2.03
Right precuneus	68	26083	9.98	-56.05	43.77	1.74
Left median cingulate and paracingulate gyri	33	15512	-5.48	-14.92	41.57	1.73
Right Calcarine fissure and surrounding cortex	44	14885	15.99	-73.15	9.40	1.63
Right cerebellum crus2	94	17038	42.12	-69.97	-45.75	1.59
Left Hippocampus	37	7469	-25.03	-20.74	-10.13	1.53
Left olfactory cortex	21	2262	-8.06	15.05	-11.46	1.50
Left lingual gyrus	47	16932	-14.62	-67.56	-4.63	1.40
Left supramarginal gyrus	63	9907	-55.79	-33.64	30.45	1.31
Right middle frontal gyrus, orbital part	10	8057	33.18	52.59	-10.73	1.27
Left angular gyrus	65	9313	-44.14	-60.82	35.59	1.15
Left precuneus	67	28358	-7.24	-56.07	48.01	1.09
Left caudate nucleus	71	7682	-11.46	11.00	9.24	1.08
Left temporal pole: superior temporal gyrus	83	10228	-39.88	15.14	-20.18	0.96
Left thalamus	77	8700	-10.85	-17.56	7.98	0.88
						

These brain regions, indexes and volumes are based on AAL116 templates, the value of weight represents the weight ratio of this brain region.

### Relationship between structural changes and cognitive behaviors

**Figure 4:**
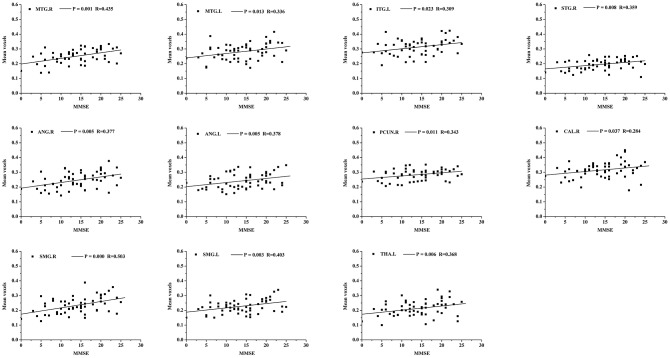
**Scatterplot of mean voxels of the bilateral MTG, bilateral ANG, bilateral SMG, left ITG, right STG, right PCUN, right CAL and left THA plotted against MMSE scores (*p*<0.05, with age, gender and education level as covariates).** MTG, middle temporal gyrus; ANG, angular gyrus; SMG, supramarginal gyrus; ITG, inferior temporal gyrus; STG, superior temporal gyrus; PCUN, precuneus; CAL, calcarine fissure and surrounding cortex; THA, thalamus.

In the AD group, positive correlations were found between the MMSE scores and the structural changes of several regions obtained from GSplit LBI (i.e., bilateral middle temporal gyrus (MTG), bilateral angular gyrus (ANG), bilateral supramarginal gyrus (SMG), left inferior temporal gyrus (ITG), right superior temporal gyrus (STG), right precuneus (PCUN), right calcarine fissure and surrounding cortex (CAL), and left thalamus (THA)) (*P* < 0.05, with age, gender and education level as covariates). The details are shown in Figure 4.

### Cross-test results on the ADNI dataset and in-house dataset

**Figure 5:**
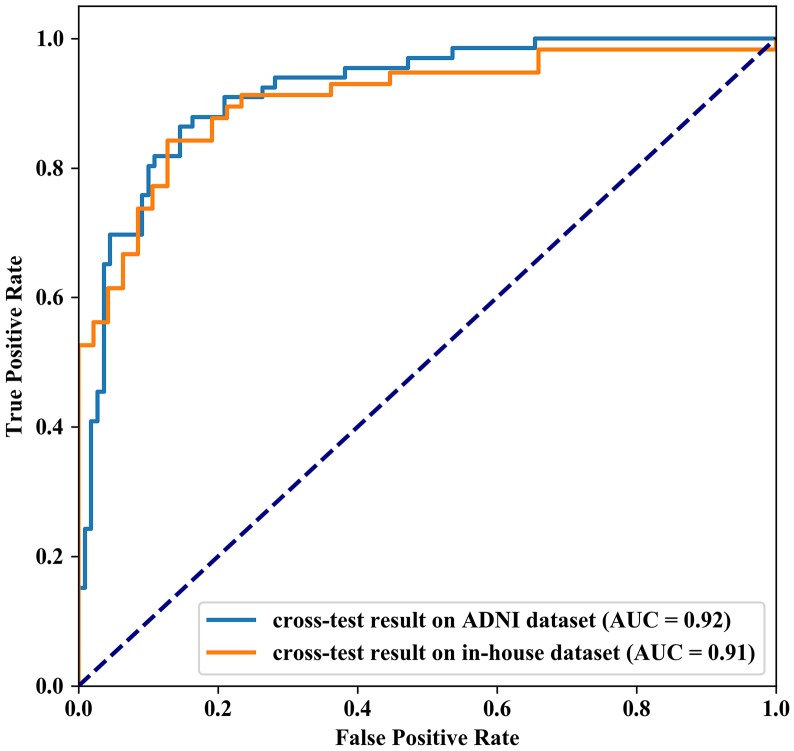
**Receiver Operating Characteristic curve of the cross-test results.** The AUC value of cross-test results on ADNI dataset and in-house dataset are 0.92 and 0.91 respectively.

In cross-test, using the Chinese model trained from our in-house dataset, the accuracy, specificity and sensitivity of the prediction results on the ADNI dataset reached 86.36%, 80.30% and 90.00%, respectively. Simultaneously, using the ADNI model, the accuracy, specificity and sensitivity of the prediction results on our in-house dataset also reached 84.26%, 78.95% and 91.49%, respectively. The ROC curve of the cross-test is shown in Figure 5, and the AUC values of these two tests were 0.92 and 0.91, respectively. When using the model trained from our in-house dataset on the ADNI dataset, the brain regions with the largest weight ratios on the classification of AD patients and NCs are shown in Figure 3B.

## DISCUSSION

Effective and accurate AD diagnosis is critical for early treatment. Therefore, many researchers have devoted their efforts to develop a computer-aided system that can diagnose AD in the early stages and on an individual basis [2, 3]. The present study demonstrated that patients with AD could be distinguished from NCs using a classified model based on GM voxels and the GSplit LBI algorithm, with good to excellent accuracy. This classification was driven by a distributed pattern of GM voxel alterations, involving the lateral temporal lobe, entorhinal cortex, hippocampus, parahippocampal gyrus, limbic system, M1, cerebellum crus2 and thalamus. In addition, we found that atrophy of several specific regions significantly correlated with cognitive performances as measured by MMSE. Finally, we performed a cross-test to verify the generalization of the model prediction and stability of feature selection in the classification.

### Advantages of GSplit LBI for AD prediction

Multiple GM microstructural abnormalities in AD have been reported in previous studies, which have enabled the discrimination of AD from NC [4–7, 10, 31–33]. However, these studies only reported group-level differences of various brain structures and did not consider evaluations of single subjects [34]. Here, we used GSplit LBI to examine whether the GM microstructural abnormalities could be used to discriminate between AD patients and NCs at the individual level. In voxel-based neuroimage analysis, lesion features have been the main focus in disease prediction due to their interpretability with respect to the related diseases. However, the “Procedural Bias”, which could be leveraged to improve classification accuracy, was introduced during the preprocessing steps [35]. Among most existing models, the models with a general loss and L2 penalty are poorly interpretable and are easy to overfit. These kinds of models automatically select or extract features that are strongly related to classification according to the principle of minimizing classification errors. Other kinds of models, such as L1 regularization-based methods, have structural sparsity and interpretability, but it is easy to ignore the effect of procedural bias on AD/NC classification. In the preliminary study of our team members [30], empirical experiments were evaluated on the ADNI dataset. The advantage of GSplit LBI is verified by the improved stability of selected lesion features and better classification results when compared with other models, such as MLDA, SVM, Lasso, Graphnet, Elastic Net, TV+l1 and n2GFL. In the present study, we used GSplit LBI to distinguish AD from NC at the individual level in a Chinese cohort, and the average accuracy, sensitivity and specificity of this classifier reached 90.44%, 91.17% and 89.50%, respectively. The misclassified cases are mainly distributed in cases with low clinical dementia rating (CDR) scores. This is because the higher the CDR scores, the heavier the dementia, the easier it is to classify. Conversely, the lower the CDR scores, the lighter the dementia, the easier it is to classify errors. In addition, the AUC value of our AD/NC classification model reached 0.9, improved the stability of selected lesion features and led to better classification results. Moreover, the ROC curve shows that our model is highly sensitive and specific.

### Brain areas involved in the classification analysis

In the present study, the discrimination was based not only on atrophy of the lateral temporal lobe, entorhinal cortex, the hippocampus, parahippocampal gyrus and limbic system but also on regions of M1, cerebellum crus2 and thalamus, which are not traditionally implicated in AD. This demonstrated the capacity of GSplit LBI to detect subtle and distributed GM alterations. Previous neuroimaging studies in AD have revealed alterations in the lateral temporal lobe, entorhinal cortex, hippocampus and limbic system [4–7, 10, 31–33], reflecting different disease stages and predicting the progression from MCI to AD [8]. In addition, AD patients accumulated abnormal proteins (Aβ and tau) in the form of amyloid plaques and neurofibrillary tangles, eventually resulting in loss of neurons in these areas [8, 36]. Alterations within these areas might explain memory problems, including difficulties in word finding and thinking processes, abilities to reason, making judgments, communication and dealing with daily activities, which are the most common symptoms in AD [37, 38]. Consistent with these previous structural studies, our findings of the high discriminative values on the lateral temporal lobe, entorhinal cortex, hippocampus and limbic system provided new evidence that microstructural abnormalities within these areas were critically occurred in AD.

Except for the common GM atrophy areas, we also found that cerebellum crus2, M1 and thalamus contributed to the identification of AD patients. This result was consistent with other AD-related studies, which were also involved in cerebellar subregions. For instance, GM atrophy of the cerebellum has been detected in AD in several neuroimaging studies [39, 40]. Furthermore, AD pathological changes have now been revealed in the cerebellum, including deposits of amyloid-b plaques, neurofibrillary tangles, and increased microglia [41–43]. As the core region, the cerebellum has efferent and afferent fibers between the vermis, hypothalamus and limbic system [44]; thus, the alteration of the cerebellum might reflect abnormal multimodal functions. In our study, we also found that M1 contributed to the identification of AD from NC. In previous studies, some task-related functional magnetic resonance imaging (fMRI) studies have demonstrated reduced activation in the premotor cortex in AD patients when performing motor-related tasks [45, 46]. In addition, one resting state fMRI (rs-fMRI) study reported the significant functional abnormality of sensorimotor (SMN) cortex in AD patients [47]. In conjunction with the previous reports and our findings, we speculated that AD patients might present subtle motor impairment caused by the atrophy and dysfunction of the sensorimotor cortex. In the present study, we found that thalamus atrophy contributed to the identification of AD patients, which was consistent with several AD related studies. Reduction of thalamic volume was typically observed in some cases of amnestic MCI [48–51]. In 2010, de Oliveira performed a longitudinal analysis of thalamic tissue texture, finding a stepwise decline in thalamic status going from controls to amnestic MCI and AD cases [52]. Likewise, thalamic volume was found to be correlated with cognitive performance in MCI patients through MRI-based measurements [50, 51]. Furthermore, reduced thalamic volume was found in AD cases when compared to other non-dementing individuals who reported memory lapses [53], with thalamic volume again correlating with the decline of global cognitive performances.

### Relationship between structural changes and cognitive behaviors

In addition, our study found a close relationship between cognitive impairment (MMSE scores) and mean voxels of several regions obtained from GSplit LBI in AD patients, including bilateral MTG, bilateral ANG, bilateral SMG, left ITG, right STG, right PCUN, right CAL and left THA, suggesting that structural changes in these regions could be used as imaging markers for tracking disease progression. Among these brain regions, the left MTG and left ITG account for a large weight on the classification between AD patients and NCs of GSplit LBI, further verifying the generalization of this model prediction and the stability of feature selection.

### Reproducibility and Stability of GSplit LBI in different cohorts

In the cross-test on our in-house dataset and the public ADNI dataset, whether using the Chinese model or the ADNI model, we successfully achieved high accuracy, sensitivity and specificity. In addition, the AUC values of these two tests were 0.92 and 0.91 respectively, indicating that our GSplit LBI algorithm not only has good performance in a local cohort but also has generalization of model prediction in different cohorts. Moreover, during the cross-test on the ADNI dataset and our in-house dataset, when using the Chinese model, the brain regions that had the greatest impact on the classification between AD patients and NCs were mainly involved in the lateral temporal lobe, entorhinal cortex, hippocampus and limbic system and cerebellum crus2, which were highly consistent with the results when using the model parameters of this experiment on our in-house dataset, further suggesting the good reproducibility and stability of feature selection of GSplit LBI.

### Future considerations

Several issues should be considered. First, these results were based on a relatively small sample size, although it provided preliminary support for the potential of GSplit LBI as a diagnostic aid for AD. In the future, large samples might be collected to confirm the results. Second, to explore whether the application of GSplit LBI could discriminate AD patients in different stages, future studies will add more samples of early stages of AD patients, such as MCI, as well as ApoE 4 carriers. In fact, in some previous studies [54–56], the author used different machine learning algorithms to classify stable MCI (sMCI) and progressive MCI (pMCI) in the ADNI dataset and achieved good results. We are currently collecting MCI patients and are performing follow-up. When the sample size is sufficient, we intend to test the GSplit LBI algorithm's classification effect on sMCI and pMCI in the in-house and ADNI datasets in future research. Third, considering the time complexity and space complexity of the algorithm, we currently only perform experiments on coarse scale voxels 8x8x8 mm^3^ in size. In the future, some finer scale voxels will be extracted and other faster optimization methods will be introduced to our experiments. Finally, in this study, we only focus on the structural changes in early AD; future work will combine multimodal neuroimaging findings, such as structural, functional and perfusion MRI, to examine whether this combination analysis could lead to higher levels of diagnostic accuracy.

## CONCLUSIONS

In summary, the present study revealed special patterns of GM abnormalities in patients with AD. By using the GSplit LBI algorithm, which has been proven to have good generalization, these GM abnormalities can be applied to accurately differentiate AD patients and NCs at the level of the individual.

## MATERIALS AND METHODS

### Participants

A total of 104 right-handed subjects participated in this study after providing written informed consent, including 57 patients with AD and 47 NCs. This study was carried out in accordance with the recommendations of the Medical Research Ethics Committee of Xuanwu Hospital. All subjects provided written informed consent in accordance with the Declaration of Helsinki. The AD subjects were recruited from patients who had consulted the memory clinic at Xuanwu Hospital for memory complaints. The NCs were recruited from the local community.

All participants underwent complete physical and neurological examinations, standard laboratory tests and neuropsychological assessments. The neuropsychological examinations included MMSE and CDR. The AD patients fulfilled the new research criteria for possible or probable AD [57, 58]. The new criteria emphasized the clinical history, neuropsychological assessment, sMRI, positron emission tomography (PET), and cerebrospinal fluid (CSF) examinations. Based on the new criteria, we carefully evaluated our study samples and confirmed each patient as AD. Among the 57 AD patients, 33 patients had a CDR [59] score of 1 and were thus assigned to a mild AD category. Additionally, 24 patients had a CDR of 2 or 3, suggesting moderate or severe AD categories.

The NCs met the following criteria: a) no neurological or psychiatric disorders, such as stroke, depression and epilepsy; b) no neurological deficiencies, such as visual or hearing loss; c) no abnormal findings, such as infarctions or focal lesions, in conventional brain MRI; d) no cognitive complaints; and e) CDR score of 0.

Participants with contraindications for MRI, such as pacemaker, cardiac defibrillator, implanted material with electric or magnetic systems, vascular clips or mechanical heart valve, cochlear implant or claustrophobia were excluded. In addition, patients with a history of stroke, psychiatric diseases, drug abuse, severe hypertension, systematic diseases and intellectual disability were excluded.

At last, we intend to briefly explain why we chose only right-handed subjects. First, the ratio of left-handed and right-handed people in the world is about 3:17. No matter in AD or normal people, samples of right-handed people are easier to collect. Indeed, most of the patients we collected were right-handed. In order to match the AD group, we selected the normal group with right-handed. Second, the size of the brain is different between the left-handed and right-handed people. Compared with the left-handed people, the right-handed people have smaller brain [60]. Therefore, it is necessary to consider the hand when quantifying the variability among individuals. Previous studies [61, 62] have consistently shown that people with smaller brain have a relatively higher percentage of gray matter, which can be used in most tissues to process local information. In this study, we focused on gray matter voxels, so only the right-handed subjects were selected.

### MRI acquisition protocol

MRI data acquisition was performed on a SIEMENS verio 3-Tesla scanner (Siemens, Erlangen, Germany). The subjects were instructed to hold still, keep their eyes closed and think of nothing in particular. 3D T1-weighted magnetization-prepared rapid gradient echo (MPRAGE) sagittal images were obtained with the following parameters: TR/TE/TI/FA = 1900 ms/2.2 ms/900 ms/9°, image matrix = 256×256, slice number = 176, thickness = 1 mm.

### Preprocessing of structural MRI data

Preprocessing of the MRI data was carried out using SPM8 software (http://www.fil.ion.ucl.ac.uk/spm) and the VBM8 toolbox (http://dbm.neuro.uni-jena.de/vbm). First, all 3D T1-weighted images were corrected for the bias field with regard to homogeneity in the VBM8 toolbox. Second, the corrected images were normalized and then segmented into GM, white matter (WM), and cerebrospinal fluid (CSF) components. Only GM components were considered in the current study. The normalization method we used was DARTEL [63], and the template we selected was the default DARTEL standard template. In the present preprocessing of structural MRI data, images were not smoothed. When no smoothing is employed, the sensitivity is increased. In addition, our model added the clustering constraints, which has its own denoising properties. After this processing, the total number of voxels is 121x145x121, and the voxel size is 1.5x1.5x1.5 mm^3^. Third, a downsampling processing was used to change the voxel size to 8x8x8 mm^3^ and the number of voxels is 24x28x24. Finally, a total number of 2,527 voxels with average values in the GM population template greater than 0.1 was extracted from the processed images and served as the input features.

### GSplit LBI-based classifier

To achieve accurate classification of AD patients and NCs, we adopted the GSplit LBI method, combining logistic regression and sparse regularization of structure, and used the generalized image descent method to optimize the parameters of such binary classification problems. The model loss function was defined as follows:

$$\begin{array}{l} ℒ\left(\beta ,\gamma \right)=-\overset{N}{\underset{i=1}{\sum }}[y_{i}*\log\left(\sigma \left(x_{i}\beta \right)\right)+\left(1-y_{i}\right)\\ \;\;\;\;\;\;\;\;\;\;\;\;\;\;\;\;\;*\log\left(1-\sigma \left(x_{i}\beta \right)\right)]\\ \;\;\;\;\;\;\;\;\;\;\;\;\;\;\;\;\;+\frac{1}{2\nu }{\left\|D\beta -\gamma \right\|}_{2}^{2} \end{array}$$

β is the main parameters of the model, γ is sparse constraint parameter for β, σ(·) is sigmoid function, and ν is a hyperparameter to balance loss term and regularization term. To obtain a better regularization path, we adopted the idea of variable splitting and introduced an additional sparse parameter γ in the regularization parameter. The structural sparsity of the model can be satisfied by controlling the square of 2-norms of sparse γ and *Dβ*. The goal of optimization is to minimize this loss function based on training set in each iteration. For more detail of Split LBI, please refer to the previous study [30].

### Design of classification experiments

**Figure 6:**
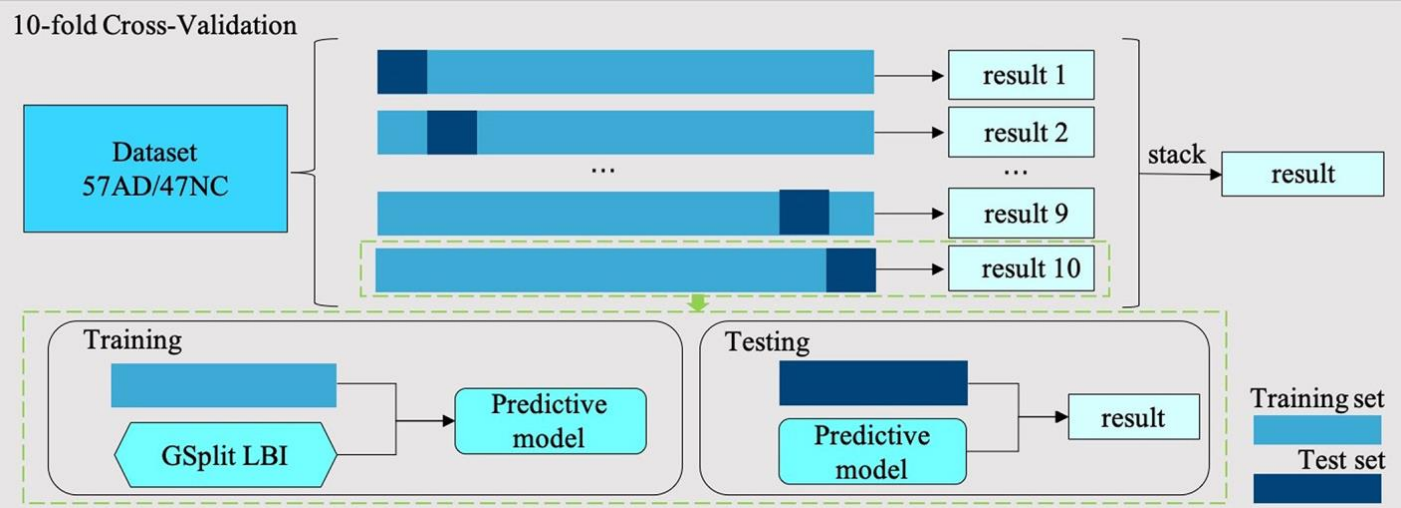
**10-fold cross-validation flow chart. In each cross-validation step, the dataset with 57 AD samples and 47 NC samples is divided into ten subsets, nine of which are used as training set and the rest as test set.** In the training step, we use the training set training GSplit LBI model. In the test step, we use the trained model to predict the test set. Finally, the results of ten folds are stacked together as the results of this 10-fold cross-validation step.

To make full use of the available data, a 10-fold full cross-validation method was used to train our model. In each cross-validation step, to ensure that all data were involved in the training and validation process, the dataset was randomly divided into ten subsets, nine of which were used to train the model in turn; after that, the trained model was used to predict the results on the remaining subset. After all the subsets had been predicted, we combined the results of the ten subsets and got the prediction results for the entire dataset, shown in Figure 6. We then calculated the model’s accuracy, sensitivity, specificity and other indicators on the results. We repeated this process 10 times and calculated the mean value and 95% CI range of these experimental results. In addition, the ROC analysis, which is a plot of the fraction of true-positive test results (sensitivity) versus the fraction of false-positive test results (1-specificity), was plotted after these experiments. Finally, the AUC was calculated to evaluate the model’s classification performance of AD patients and NCs, and the mean and 95% CI of the ROC curve were used to evaluate the stability of the model.

### Brain area analysis in the classification

We carried out a sensitivity analysis of our classification model by analyzing which parts of the gray matter voxels have greater impacts on the classification between AD patients and NCs. In this analysis, we take the parameters corresponding to each voxel in main parameter β as its contribution to classification. The weight parameters in parameter β was sorted from large to small and then performed the classification experiments with the largest top n (range from 1 to 2527) weight parameters in turn. The relationship between the prediction accuracy and the number of weight parameters used in the prediction was then recorded in a chart. When the accuracy reached the maximum and remained unchanged, we obtained all the key voxels that affected the prediction results.

In addition, we constructed a brain region weight map based on the Automated Anatomical Labeling (AAL) [64] atlas using the following steps. First, we mapped the weight parameters to their corresponding voxels on a DARTEL template and obtained a coarse weight distribution map with voxel size 8x8x8 mm^3^. Second, the coarse weight distribution map was transformed to a finer weight distribution map with voxel size 1x1x1 mm^3^ by the nearest-neighbor interpolation. This finer weight distribution map had the same dimensions as the AAL template. Third, we mapped the finer weight distribution map to the AAL template by multiplying the corresponding elements and calculated the sum of weight values in each brain region. These values were defined as the weight value of each brain region. Finally, according to the weight value of each brain region in AAL, we constructed a brain region weight map and calculated the relative weight ratio of each brain region.

### Correlation analysis

To explore the relationships between cognitive impairment (MMSE scores) and mean voxels of several regions obtained from GSplit LBI in AD patients, a partial correlation analysis was performed, with age, gender and education being used as nuisance covariates (SPSS20, *P* < 0.05).

### Reproducibility and Stability analysis

To verify the generalization of model prediction and the stability of feature selection, we performed a cross-test on our in-house dataset and the public ADNI dataset. We used the model parameters trained from this experiment to test the ADNI dataset (to facilitate the reference later, here we name it the Chinese model); we also used the model parameters trained from the ADNI dataset of our previous work [30] to test the current dataset (to facilitate the reference later, here we name it the ADNI model).

In this cross-test experiment, we used the same ADNI subjects as our previous work [30] In the ADNI dataset, all subjects are divided into 1.5 Tesla and 3.0 Tesla MRI scan datasets. For comparison, we chose the 3.0T ADNI dataset in our experiment, which contains a total of 176 subjects (66 AD patients and 110 NCs) with ages ranging from 55-90 years old. The average educational levels of AD and NC are 15.59 and 16.65 respectively; meanwhile, the average of MMSE scores for AD and NC are 23.11 and 28.93, respectively. Subject IDs are shown in the Supplemental material in our previous paper [30].

## Supplementary Material

Supplementary Figure 1

Supplementary Tables
