# Supplementary material for: Application of Generalized Split Linearized Bregman Iteration algorithm for Alzheimer's disease prediction
Source: Aging (Albany NY). 2020 Apr 5;12(7):6206–24. doi: 10.18632/aging.103017 (PMC7185109; doi:10.18632/aging.103017)
Supplement: Supplementary Figure 1 [file aging-12-103017-s002..pdf]

## SUPPLEMENTARY FIGURE

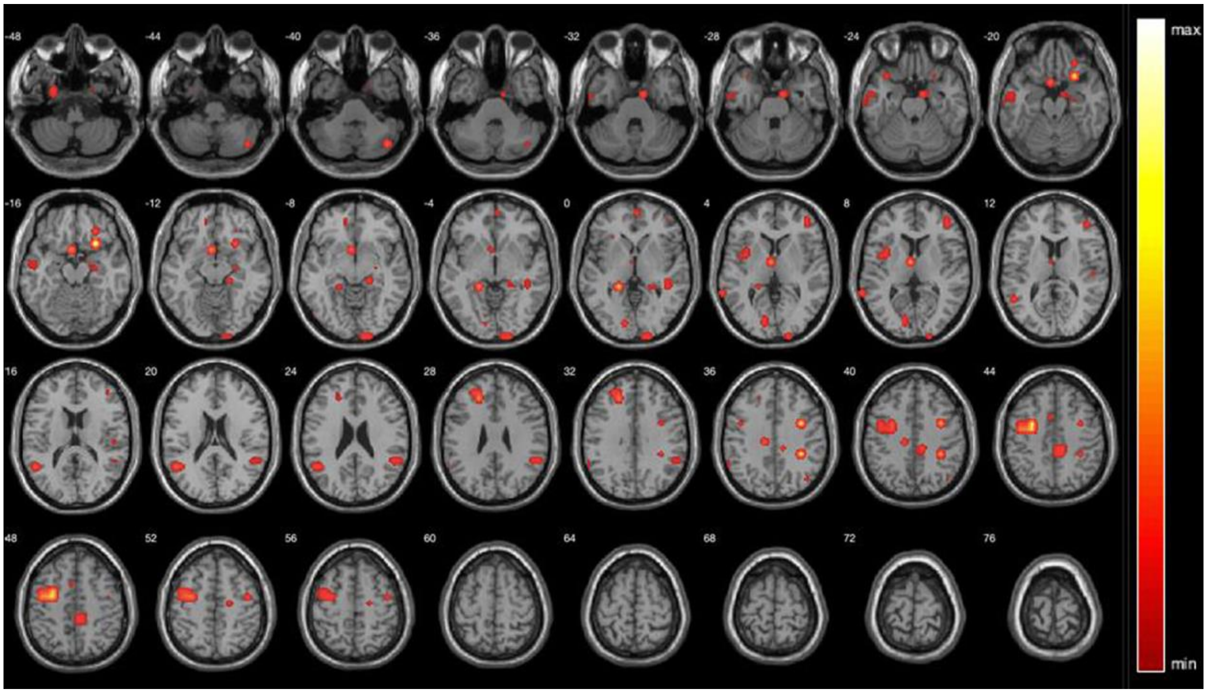


**Supplementary Figure 1. Weight distribution map to classification between AD patients and NCs which is reconstructed by combining voxel information that is entered into the model and top 6% of the model parameters.** The color bar represents the weight value form GSplit LBI model, the larger the weight value of the model, the warmer the color in the graph.
