# Supplementary material for: Application of Generalized Split Linearized Bregman Iteration algorithm for Alzheimer's disease prediction
Source: Aging (Albany NY). 2020 Apr 5;12(7):6206–24. doi: 10.18632/aging.103017 (PMC7185109; doi:10.18632/aging.103017)
Supplement: Supplementary Tables [file aging-12-103017-s001..pdf]

SUPPLEMENTARY TABLES

Supplementary Table 1. Performance (accuracy, sensitivity, specificity) of ten experiments, each experiment is a ten-fold cross-validation.

	accuracy	sensitivity	specificity
1st	90.45%	91.67%	88.50%
2nd	89.55%	91.00%	87.50%
3rd	91.36%	91.67%	92.00%
4th	90.45%	91.67%	90.00%
5th	90.45%	91.00%	89.50%
6th	90.55%	91.00%	89.50%
7th	89.55%	91.33%	87.00%
8th	90.45%	89.67%	91.50%
9th	91.36%	91.33%	91.00%
10th	90.27%	91.33%	88.50%
average	90.44%	91.17%	89.50%

Supplementary Table 2. In previous study, the GSplit LBI-based classifier had been compared with other classifiers including MLDA, SVM, Lasso, Graphnet, Elastic Net, TV+l1 and n^2GFL and obtained better results than other models on ADNI dataset.

	SVM	Lasso	Elastic Net	MLDA	Graphnet	TV+l1	n^2GFL	GSplit LBI
ADNI	87.50%	87.50%	89.2%	86.93%	88.64%	87.50%	87.50%	90.91%
In-house	87.20%	87.20%	89.50%	-	-	-	-	90.44%

In this study, some comparative experiments including SVM, Lasso and Elastic Net also have been done to prove the performance of the GSplit LBI-based model on our in-house dataset. The result of comparative experiments between GSplit LBI-based classifier and other classifiers on ADNI dataset and our in-house dataset are shown in this table.
